# 

**Published:** 1884-11-20

**Authors:** 


### TABLE OF CONTENTS.

ORIGINAL AND SELECTED ARTICLES:

Remarks of J. McF. Gaston, M. D., on taking the Chair of Surgery in the Southern Medical College, Atlanta, October 8, 1884, 401. Therapeutics and Materia Medica—Borate of Quinine; Tannate of Cannabin; Antipyrine; Salicylic Acid in the Treatment of Cerebro-spinal Meningitis; Some Therapeutic Uses for Lobelia; Asclepias Incarnata, 412. An Instructive Case of Vomiting of Pregnancy, by John M. Upshur, M. D., Professor of Materia Medica and Therapeutics, Medical College of Virginia, 415.

ABSTRACTS AND GLEANINGS:

Pneumonia, Bronchitis, and Pleurisy treated Antisceptically, 418. The Compass Plant, 419 The missing link of the Cholera Germ found at last; Fever and Kairin; Plethysmographic Researches, 420; Treatment of Asthma; Prevention of Ophthalmia Neonatorum, 421. The Paste Treatment of Inflammatory Skin Diseases, especially Eczema, 422. The Treatment of Pernicious Intermittent Fever, 423 Tetanus Treated with Quinine and Chloral; Treatment of Fracture of the Clevicle, 424. A new field for the Aspirator, 425. Digitalis to Prevent Excessive Desire for Copulation; Treatment of Malaria, 426. Therapeutical Remarks on Hamamelis Virginica; The New Anæsthetic, 427. Fever, 428 Typhoid Fever, 429. A New Hair Dye; Extirpation of Small Round Tumors of the Skin with a quickly rotating Punch, 430.

SCIENTIFIC ITEMS:

The oldest Living Microcephalic; A Wonderful Clock; Hygienic Sewer Arrangement, 431; Cholera Germs under the Microscope; Selenetropism, 432.

PRACTICAL NOTES AND FORMULÆ.

Treatment of Acute Rheumatism; Upham's Asthma Remedy; Cholera, 433; Quinine; Squibb's Diarrhœa Mixture, 434. Dysentery; To remove Rust Stains from Cotton and Linen; Government Harness Dressing, 435.

EDITORIALS AND MISCELLANEOUS. ;

Editorial Notices, 436. Indifference of Southern Physicians to their home journals New York Medical Society and Homœophy, 437. Death of Professor Dugas; Books and Pamphlets Received, 438. Receipted, 439; Special Notices, 440.

